# Regulatory roles of insulin growth factor binding protein family in neuroblastoma cell proliferation and differentiation: Potential prognostic biomarkers and therapeutic targets for neuroblastoma

**DOI:** 10.1002/pdi3.68

**Published:** 2024-06-08

**Authors:** Kai Huang, LinYu Yang, Yue Ma, Shan Wang

**Affiliations:** ^1^ Department of Pediatric Surgical Oncology Children’s Hospital of Chongqing Medical University National Clinical Research Center for Child Health and Disorders Ministry of Education Key Laboratory of Child Development and Disorders Chongqing Key Laboratory of Pediatric Metabolism and Inflammatory Diseases Chongqing China

**Keywords:** differentiation, growth, IGFBPs family, neuroblastoma

## Abstract

Neuroblastoma (NB), as a representative of tumors of embryonic origin in children, has specific clinical features. On the one hand, a very small number of NBs may appear to regress on their own. On the other hand, highly malignant NBs can invade the surrounding blood vessels and organs and metastasize to distant bone, bone marrow, and lymph nodes in the early stages of the disease. Based on differential affinities to insulin growth factors (IGFs), insulin growth factor binding proteins (IGFBPs) are classified into two groups: IGF binding proteins (IGFBP1‐6) with high‐affinity and IGF low‐affinity binding proteins, such as IGFBP‐related proteins (IGFBP rP1‐10). IGFBP are crucial regulators of the bioavailability and function of IGF in metabolic signaling and as modulators of IGF signaling, and their role in NB is gaining increasing attention. In this study, we investigate the involvement of IGFBP family members in the growth and differentiation of NB cells, as well as the potential of IGFBPs as prognostic biomarkers and therapeutic targets for human NB.

## 1 INTRODUCTION

Neuroblastoma (NB) is the most common extracranial solid tumor in childhood, accounting for 15% of all pediatric cancer‐related fatalities and affecting 90% of children younger than 5 years old. NB occurs in the developing sympathetic nervous system (from any neural crest component), and inhibition of differentiation of the sympathoadrenal spectrum derived from the neural crest contributes to NB formation.^1^, ^2^ Moreover, NB is highly varied, with unique but varying biology and clinical characteristics, ranging from spontaneously regressing asymptomatic benign malignancies to aggressive proliferative tumors that can spread far and are frequently fatal.^3^, ^4^ With advances of research at the molecular level recently, the mechanisms behind the development of NB become increasingly clear. Chromosomal abnormalities are extremely prevalent in NB (occurring in roughly 90% of patients); for instance, MYCN amplification on chromosome 2p24 is a well‐established driver of high‐risk NB and a major prognostic marker.^5^ MYCN is a key transcriptional regulator of cell growth, metabolism, and differentiation.^6^ Similarly, familial NBs, which account for 2%–3% of all NB cases, are associated with highly penetrant germline activation alterations in the anaplastic lymphoma kinase gene (80% of familial NBs cases) or inactivating mutations in the *PHOX2B* gene (encoding the transcription factor paired mesoderm homeobox protein 2B).^7^, ^8^ Furthermore, genetic changes impacting telomere preservation are commonly observed in patients with high‐risk NB and are associated with worse clinical outcomes. NB cells maintain telomere length through mutually exclusive processes, such as increased telomerase activity due to overexpression of telomerase reverse transcriptase (TERT) or alternative lengthening of telomeres.^9^, ^10^, ^11^ Due to clinical trials combining high‐dose chemotherapy, autologous stem cell transplantation, targeted agents, and immunotherapy with anti‐GD2 monoclonal antibodies,^12^ the 5‐year survival rate for patients with metastatic NB has increased from 20% to >50% over the past few decades. Differentiation therapy is a revolutionary treatment that reactivates the endogenous differentiation mechanism, stimulates cancer cell maturation, and returns malignant cells to a more benign phenotype. NB cells are characterized by spontaneous regression and reverse differentiation, suggesting that NB differentiation therapy is possible. Induction of differentiation may be a crucial part of direct tumor killing, and the development of differentiation modulators promises to alleviate the existing bottleneck in high‐risk resistant NB cases.^13^ In this review, we explored in considerable depth of the role that members of the insulin growth factor binding protein (IGFBP) family play in the development and differentiation of NB cells, which likely are an important therapeutic target for differentiation treatment.

## 2 NEUROBLASTOMA CELL GROWTH AND DIFFERENTIATION

### 2.1 Mechanism of growth and differentiation of neuroblastoma

#### 2.1.1 Activation of telomere maintenance mechanisms

Activation of telomerase can be triggered by amplified MYCN or TERT rearrangements in NB.^14^, ^15^ MYCN transcriptionally activates TERT in MYCN‐amplified NB tumors, resulting in telomerase activity. TERT is also transcriptionally activated by enhancer hijacking, where genomic rearrangements place the TERT gene under the control of powerful super‐enhancer elements; TERT rearrangements constitute the second route of telomerase activation in high‐risk NBs.

An active telomere maintenance mechanism is required for an indefinite proliferative potential, which can be acquired by activating the reverse transcriptase telomerase or selective telomere lengthening.^16^, ^17^ On the one hand, it is well‐established that cancer cells require telomeric DNA to sustain cell proliferation. Recent research suggests that the neural crest origin of NB may strengthen the link between telomeres status and tumor aggressiveness. In NB, telomere structure and telomerase activity are correlated with the status of adrenergic/mesenchymal differentiation, and modulation of telomerase activity can induce tumor cell differentiation.^18^ Understanding the interrelationships and effects of telomere‐based therapies on NB cell proliferation and differentiation is crucial for getting these therapies to the clinic and creating more effective techniques.

#### 2.1.2 MYCN

MYCN is overexpressed in tumors via multiple mechanisms, including induced transcriptional activation of MYCN, increased MYCN protein stabilization due to dysregulated MYCN phosphorylation, reduced proteasomal degradation, as well as MYCN gene amplification.^19^, ^20^, ^21^ Gene amplification of MYCN is one of the earliest genetic markers identified in NB and one of the strongest predictors of poor prognosis.^6^, ^22^, ^23^ Twenty to 30% of patients with NB exhibit MYCN amplification, and their overall survival rate remains below 50%. In a subtype of NB with a high risk, MYCN gene amplification is an early and likely starting event that leads to tumor formation.

MYCN is necessary for cell proliferation and migration, and lower levels have been linked to the completion of neuronal differentiation.^24^, ^25^, ^26^ It is interesting to note that inhibiting MYCN expression results in decreased proliferation and differentiation, highlighting the significance of MYC signaling in the biology of NB. For instance, in LAN‐5 NB cells, MYCN was raised 2 days after differentiation induction, and subsequently, it was predicted to be downregulated. Additionally, positive regulation of numerous differentiation markers was related to enhanced MYCN expression. Similarly, silencing MYCN was shown to hinder this differentiation, which resulted in the negative regulation of a variety of differentiation markers.

In addition, the overexpression of the MYCN gene in the NB cell line SK‐N‐AS, which had only a limited potential for differentiation, was shown to restore this capacity.^27^ Therefore, NB medication that directly targets MYCN might potentially have some promise. There is also a conceptual foundation for therapeutic interventions that target MYCN and prevent NB from differentiating into other cell types.

#### 2.1.3 Noncoding RNA

Noncoding RNAs (ncRNAs) are diverse molecules that are not translated into protein function to regulate gene expression at the transcriptional and posttranscriptional levels.^28^ miRNAs or short noncoding RNAs are single‐stranded RNAs ranging from 19 to 25 nucleotides in length. Certain microRNAs influence essential NB processes like apoptosis, differentiation, and cell proliferation.^29^ In the study of Abhishek Jauhari et al., for instance, decreased levels of Dicer caused differentiated cells to senesce, and differentiated SH‐SY5Y cells required an increase in P53 expression in response to changes in mature neuron protein levels. Regarding the role of P53 in differentiated SH‐SY5Y cells, we found that the induction of miR‐222, miR‐192, and miR‐145 was P53‐dependent.^30^

LncRNAs can function as competing endogenous RNAs, either as scaffolding for proteins or as participants in histone modifications, influencing NB proliferation, migration, invasion, and differentiation.^31^ lncRNA NB‐associated transcription factor‐1 (NBAT‐1) is a biomarker that significantly predicts clinical outcomes in NB. Loss of NBAT‐1 enhances cell proliferation and invasion and regulates these activities by silencing target genes epigenetically. Moreover, the absence of NBAT‐1 influences neuronal development by activating the neuron‐specific transcription factor REST.^32^ circRNA modulates gene expression predominantly via microRNA sponging, and dysregulation (abnormal up‐ or down‐regulation) of circRNA is implicated in a number of cancer processes, including NB. Numerous ncRNAs are dysregulated in NB, suggesting that they play a crucial role in the pathogenesis of the disease.^33^

### 2.2 Differentiation therapy for neuroblastoma

**FIGURE 1:**
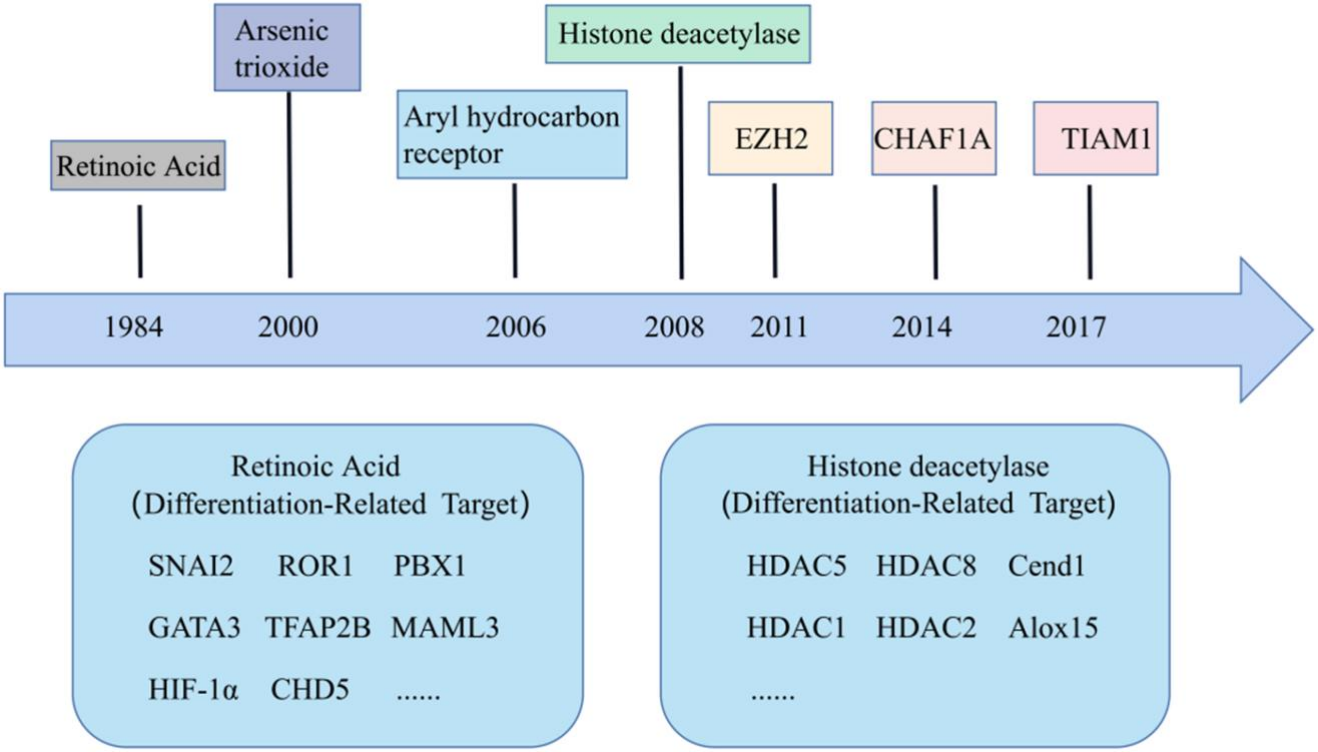
Development of differentiation therapy for NB. In 1984, the use of retinoic acid was initiated for the treatment of NB by affecting differentiation. In recent years, it has been observed that more and more differentiation‐related targets have the potential to be used in NB therapy. NB, neuroblastoma.

It can regress spontaneously and differentiate spontaneously into normal cells or benign ganglion cell tumors after medium or low dose chemotherapy.^34^ In other words, NB cells are characterized by spontaneous regression and reverse differentiation, suggesting the possibility of NB differentiation therapy. Given the aberrant differentiation of NB cells, treatment to induce NB cell differentiation has proven to be an effective therapeutic strategy, and recently, several differentiation‐related targets have been identified, including AHR, HDAC1/2, and EZH2, which provide the basis for the development of differentiation therapeutic strategies.^35^, ^36^, ^37^ aTRA, 13‐cis‐RA, and arsenic trioxide have pro‐differentiation effects in a variety of tumors and are used in clinical treatment of NB.^38^, ^39^, ^40^, ^41^, ^42^ We summarize the studies from the induction of differentiation therapy in Retinoic acid (RA) in 1984 to the latest differentiation‐related targets (Figure 1), which provide a theoretical basis for future differentiation targeting.

## 3 IGFBPs IN NB CELL GROWTH AND DIFFERENTIATION

The IGFBP sequence consists of 200–300 amino acids and three structural domains: a disulfide‐bond restricted cysteine‐rich amino‐terminal structural domain, an evolutionarily conserved cysteine‐rich carboxy‐terminal structural domain, and a structurally less structured or unstructured linkage structural domain with variable sequence among family members. IGFBPs are extensively expressed in most tissues and are adaptable endocrine, autocrine, and paracrine regulators of insulin growth factor (IGF) activity, which is crucial for these vital physiological system regulators.^43^, ^44^, ^45^ On the one hand, at the cellular level, in addition to inhibiting IGF activity through competitive binding, IGFBP enhances IGF activity through a variety of mechanisms. For example, in fibroblast cultures, IGFBP‐5 binds to components of the extracellular matrix, thereby decreasing its affinity to bind IGF‐I and increasing IGF‐I activity.^46^ In wound healing models, IGF‐I activity can be enhanced by IGFBPs.^47^ On the other hand, IGFBPs are multifunctional proteins with a large number of different intrinsic roles that do not depend on binding to IGF. For example, some studies have been reported on IGFBP‐5 and IGFBP‐3 specific cell surface receptors, although these remain to be identified or validated.^48^ The most direct role is that of IGFBP‐1 and IGFBP‐2 via cellular integrin receptors.

**FIGURE 2:**
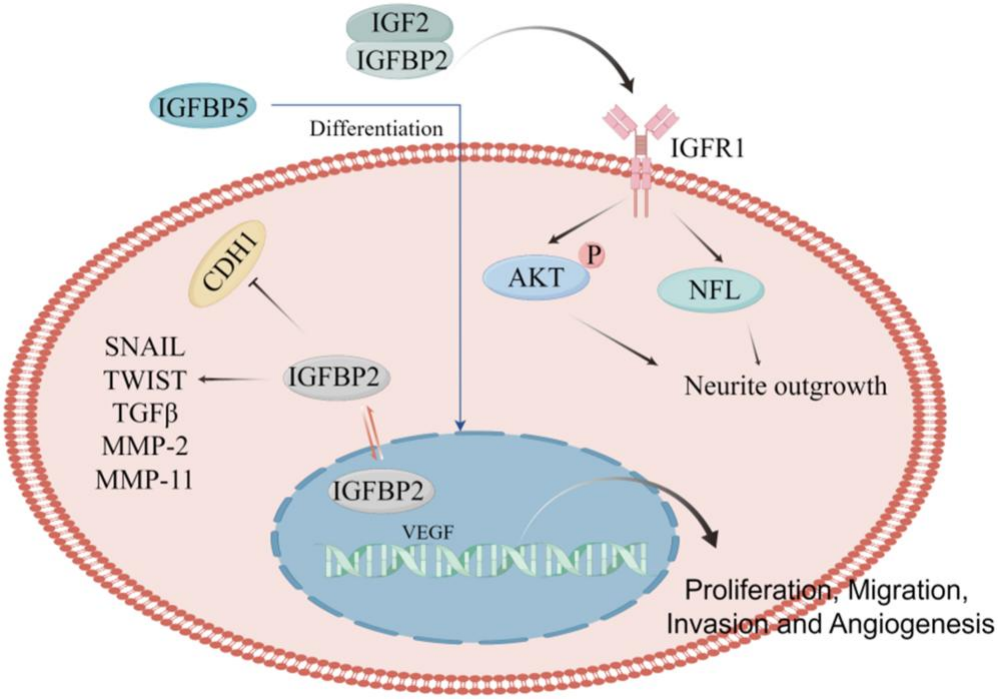
Regulatory roles of IGFBP family in NB cell proliferation and differentiation. IGFBPs can simultaneously promote tumor progression and differentiation dependents on insulin growth factor signaling or by directly acting on NB cells. IGFBP, insulin growth factor binding protein; NB, neuroblastoma.

Recent studies reported the IGFBP family in pan‐cancer, and the results proposed that the expression of IGFBP family was significantly correlated with tumor mutational load, microsatellite instability, tumor stemness, and tumor immune microenvironment. And preliminary experiments were conducted to verify that IGFBP2 and IGFBP6 were lowly expressed in gastric cancer, while IGFBP6 was lowly expressed in colorectal cancer.^49^ Similarly, another study demonstrated that the IGF/IGFBP signaling axis promotes advanced pancreatic cancer (PC) by promoting tumor progression, metastasis, and drug resistance.^50^ A vast body of evidence demonstrates that the IGF/IGFBP signaling axis promotes advanced PC by promoting tumor development, metastasis, and medication resistance. Similarly essential is the role of IGFBPs in assessing lung cancer risk, prognosis, and medication resistance. Moreover, alterations in the equilibrium of IGF system components may contribute to developing breast and gastric malignancies.^51^, ^52^ This study focused on the impact of IGFBPs in NB cell proliferation and differentiation to offer a theoretical foundation for future targeted NB therapy (Table 1, Figure 2).

**TABLE 1:** The role of IGFBPs in neuroblastoma.

Genes	Author	Year	Expression	Cell line	Effect	Ref
IGFBP2						
	V. C. Russo	2004	Up	SK‐N‐SHEP	Proliferation and metastatisis	53
	Walid J. Azar	2011	Up	SK‐N‐SHEP	Invasive	54
	Eun Young Jeong	2013	Up	SH‐SY5Y	Proliferation	55
	WJ. Azar	2013	Up	SK‐N‐SHEP	Angiogenesis	56
IGFBP‐3						
	Dongyun Zhang	2019	Down	SH‐SY5Y	‐	57
IGFBP‐5						
	Barbara Tanno	2006	Up	LAN‐5	Mitochondrial apoptosis	58
	Vincenzo Cesi	2005	Up	RN‐GA	Differentiation	59
	Barbara Tanno	2002		LAN‐5	Proliferation	60
	Vincenzo Cesi	2004	Up	SK‐N‐AS, SK‐N‐BE, SK‐NBE2(C)	Proliferation	61
IGFBP‐6						
	S. Babajko	1997	Down	SH‐SY5Y	Proliferation	62
	D. Seurin	2002	Down	SK‐N‐SH	Proliferation	63

Abbreviation: IGFBP, insulin growth factor binding protein.

### 3.1 IGFBPs in NB cell growth

IGFBP2 has been extensively studied as a secreted protein or cytoplasmic signaling effector in cancer cells in many malignant cancers.^64^ Involved in important cell signaling regulatory molecular interactions, these molecules are involved in phosphatase and tensin homolog regulation, EGFR/STAT3 regulation, and NF‐kB activation. In human NB cell lines, IGFBP‐2 enhances the proliferation and invasion of adult NB cells and is also an activator of invasive behavior in cancer cells.^53^ Involved in the activation of nuclear entry and native gene expression programs, including transcriptional regulation of VEGF genes and subsequent proangiogenic activity in xenograft NB cells in vivo, nuclear IGFBP‐2 is required for the activation of VEGF expression and subsequent angiogenesis.^54^ At the same time, IGF‐2 and IGFBP2 synergistically increased neuro‐protrusion growth through enhanced early signaling of IGF‐1 type receptors. In addition, bone glycine, a small leucine‐rich proteoglycan, was found to significantly increase IGF‐2/IGFPB2‐induced neuronal axon growth.^55^

As in other components of the IGF system, the role of IGFBP6 has been studied in a considerable number of cancer cells and models.^65^, ^66^, ^67^ In most studies, IGFBP‐6 expression was lower in malignant cells than in normal cells, suggesting that it has an inhibitory effect.^68^ IGFBP‐6 expression is consistent with a reduced proliferative responsiveness of NB cells to IGFs and insulin.^62^ Constitutive IGFBP‐6 overexpression inhibits NB xenograft growth in vivo.^69^ In addition, infusion of IGFBP‐6 may also retard NB xenograft growth in vivo by inhibiting the action of IGF‐II.^63^

### 3.2 IGFBPs in NB cell differentiation

NB consists of NB cells and undifferentiated Schwann cell precursors, which provide an opportunity for pro‐differentiation and antiproliferative therapy in high‐risk NB^1^; we have focused more on the differentiation‐related role of IGFBPs in NB. According to the literature, IGFBP5 is upregulated in mesenchymal stem cells present in craniofacial tissues to enhance differentiation and periodontal tissue regeneration, and has a neurogenic differentiation potential for promoting directed differentiation of dental pulp stem cells.^70^ Similarly, RA‐induced differentiation in NB results in a dramatic increase in IGFBP‐5. Functional assays performed under differentiation conditions showed that IGFBP5 transcription was sensitive to RA treatment.^59^ Another report investigated the relevance and function of endogenous IGFBP‐5 in LAN‐5 and SY5Y(N) cell lines, where the ability of these cells to undergo neuronal differentiation was impaired following IGFBP‐5 inhibition, but this effect was reversed by exposure to recombinant IGFBP‐5.^71^ In addition, CgA is a tissue‐specific protein restricted to the diffuse neuroendocrine system and is widely expressed in NB. Deletion of CgA decreased IGF‐II and IGFBP‐2 expression, increased IGFBP‐3 levels, and inhibited IGF downstream signaling, and in an in‐vivo heterogeneous NB model, CgA knockdown resulted in increased protein and mRNA levels on *s* increased expression of phenotypic markers. These results suggest that CgA maintains IGF secretion and intracellular signaling pathways to regulate the proliferation and differentiation of adult NB.^57^

## 4 CONCLUSION

IGFBPs have been recognized as cancer biomarkers in several human cancers, and recent advances in the understanding of differentiation‐related IGFBPs in tumors have contributed to molecularly targeted tumor therapy. NB arises as a result of a differentiation block in the developing sympathetic adrenal spectrum, including adrenergic and nonneuronal mesenchymal cell populations with heterogeneous tumor cells whose cellular phenotype may resemble some aspects of normal developmental heterogeneity. Recent sc/snRNA‐seq studies have further refined this developmental heterogeneity and provided a basis for understanding their genealogical relationships and ability to interconvert.^72^, ^73^, ^74^ Whether IGFBPs have a mechanism of action during divine mother differentiation needs further elucidation and in‐depth study.

## AUTHOR CONTRIBUTIONS

Kai Huang reviewed the literature and prepared the figure. Kai Huang and LinYu Yang wrote the manuscript. Yue Ma provided language help and writing assistance. All authors were involved in the conception, preparation of the manuscript, and the final version of the manuscript has been read and approved by all the authors before its submission.

## CONFLICT OF INTEREST STATEMENT

The authors do not report any conflict of interest.

## ETHICS STATEMENT

None.

## Data Availability

The data that support the findings of this study are available from the corresponding author upon reasonable request.
